# *Candidozyma auris* and carbapenemase-producing organisms travel screening: program implementation and initial report

**DOI:** 10.1017/ash.2026.10871

**Published:** 2026-10-09

**Authors:** Katelyn West, Nicole Russo, Christopher D. Pfeiffer

**Affiliations:** 1 Department of Hospital and Specialty Medicine, VA Portland Health Care System, Portland, OR, USA; 2 Department of Medicine, Oregon Health and Science University, Portland, OR, USA

## Abstract

*Candidozyma auris* and carbapenemase-producing organisms pose emerging healthcare threats. We implemented a travel-based screening program at a VA hospital. Among 3,199 admissions, one patient tested positive for a carbapenemase-producing organism, and no *Candida auris* cases were detected. Implementation revealed operational challenges, including prolonged laboratory turnaround times and workflow barriers.

## Background

*Candidozyma auris (C*. *auris)* and carbapenemase-producing organisms (CPOs) are rapidly emergent healthcare-associated pathogens with substantial morbidity, mortality, and transmission risk. Early identification and isolation of colonized patients are essential to limiting healthcare-associated spread.^1,2^ In Oregon, both organisms remain uncommon; therefore, local public health guidance emphasizes targeted, travel-based screening for regional prevention.^3,4,5^ In 2024, the VA Portland Health Care System (VAPORHCS) implemented a *C. auris*/CPO screening program as a quality improvement initiative.

## Methods

For multidrug-resistant organism (MDRO) surveillance, in addition to electronic health record (EHR) infection control flags, we used VA Bug Alert (VABA), a surveillance software program that alerts subscribed personnel to newly admitted patients with a previously detected MDRO anywhere in VA nationally.^6^

Beginning April 1, 2024, all acute care admissions were screened using the standardized question: “Have you had an overnight stay in a hospital, nursing home, or other healthcare facility outside of Oregon or Washington in the last year?” Responses were documented in the nursing admission transfer note.

Patients answering affirmatively were placed in private rooms, and screening orders were initiated by the bed flow coordinator. After verbal consent, nurses collected bilateral axilla/groin swabs for *C. auris* and peri-rectal swabs for CPOs. *C. auris* specimens were processed at West Regional Antimicrobial Resistance Laboratory Network (ARLN) laboratory (Shoreline, WA); CPO testing was performed in-house using multiplex PCR (Xpert® Carba-R). Turn-around-time (TAT) was recorded.

**Figure 1.**
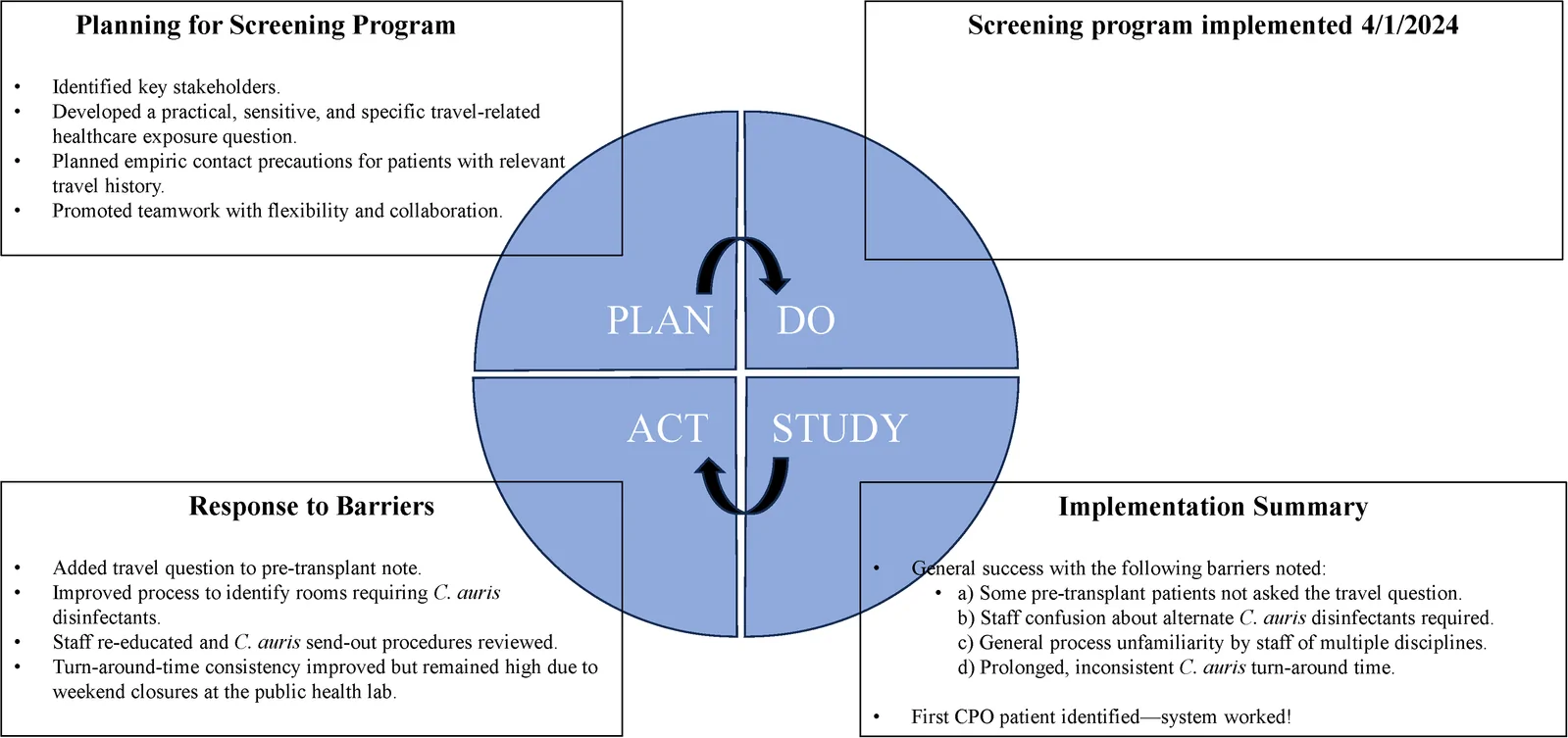
Plan-Do-Study-Act (PDSA) framework used to implement *C. auris/* CPO screening.

Empiric contact precautions were initiated pending results, and enhanced environmental cleaning protocols were used for suspected *C. auris.* ^7^ Implementation was guided by a Plan-Do-Study-Act (PDSA) framework, which allowed the team to systematically identify and address operational barriers, including missed screenings, staff re-education needs, communication gaps, complex disinfection protocols, and unanticipated delays in *C. auris* test reporting (see Figure 1). Retrospective chart review was performed to validate reported travel exposures.

## Results

Between 4/1/2025 and 11/30/2024, 3,199 acute care admissions occurred. Seventy-two patients (2.3%) reported qualifying travel-related healthcare exposure. Of these, 64 reported exposures within the United States or U.S. territories (including Puerto Rico, n = 5), and 8 reported international exposure (Mexico, n = 6; Philippines, n = 2).

Nine patients (12.5%) were not tested due to refusal (n = 2), staff assessment (n = 3), readmission (n = 1), or technical issues (n = 3). An additional 32 patients initially reported qualifying travel that was not substantiated on chart review; 16 of these underwent screening, all with negative results.

Mean TAT for *C. auris* and CPO testing was 7.7 days **(**range, 3–18 d) and <1 day, respectively.

No *C. auris* colonization was detected. One patient (2.4%) tested positive for CPO; this case was also identified by VABA.

## Discussion

We identified ∼9 patients monthly with qualifying travel-related healthcare exposure in our ∼100-bed tertiary VA hospital, including ∼1.6 patients monthly with exposure outside the 50 U.S. states.

Identification of a single CPO-colonized patient on admission was meaningful; subsequent clinical cultures were CPO-positive such that early detection prevented a contact investigation (as is standard in our jurisdiction) and potentially an outbreak. The positive travel screening questionnaire and subsequent rapid CPO identification on admission prompted immediate contact precautions. Although VABA also detected this case soon after admission due to prior colonization, VABA requires engagement of subscribed local facility personnel and does not identify incident and previously detected non-VA cases.

Several operational challenges emerged. EHR limitations initially excluded certain admission types (ie, pretransplant) requiring informatics modifications. Communication gaps between infection prevention and clinical teams led to premature discontinuation of empiric precautions, underscoring the need for continued education. To improve adherence, visual cues placed outside patient rooms were introduced to indicate *C. auris*-specific environmental cleaning requirements, improving interdisciplinary coordination. Consideration was given to routine use of EPA-registered disinfectants active against *C. auris* but concerns regarding corrosiveness prevented adoption during the study period.^8^

Prolonged *C. auris* TATs were driven by laboratory staffing limitations and weekend/holiday processing delays. Although bed flow was not impacted, the prolonged empiric contact precautions, often for the duration of hospitalization, created a larger-than-anticipated number of days in isolation with downstream effects such as increased gown and glove consumption alongside the potential for higher levels of patient depression and anxiety.^9^ While testing was free through public health, other program costs included personnel time.

The implementation has been successful. The program benefits include the early detection of targeted MDROs to prevent outbreaks for travel-related exposures with relatively low operational impact. The screening question prioritized sensitivity over specificity, consistent with local public health recommendations.^3,5^ Future directions include more accurate identification of higher-risk exposures (eg, international, long-term acute care), evaluation of in-house *C. auris* testing to reduce TAT, and thoughtful consideration of additional ways to reduce days and impact of contact precautions.

## Conclusion

The *C. auris/*CPO screening program was successfully implemented and identified one CPO case. The PDSA framework facilitated structured implementation and collaborative problem-solving. A low detection rate and long *C. auris* laboratory TAT represented important limitations, emphasizing the need for ongoing program evaluation.

## Previous presentations

This work was presented in part at SHEA Spring 2025, Abstract #307.
